# Prenatal and postnatal correlates of moderate-to-vigorous physical activity in midlife: evidence from the 1970 British Cohort Study

**DOI:** 10.1136/jech-2022-219213

**Published:** 2022-09-09

**Authors:** Joanna M Blodgett, Thomas Norris, Emmanuel Stamatakis, Gary O'Donovan, Snehal M Pinto Pereira, Mark Hamer

**Affiliations:** 1 Institute of Sport Exercise & Health, Department of Targeted Intervention, Division of Surgery & Interventional Science, University College London, London, UK; 2 Charles Perkins Centre, School of Health Sciences, Faculty of Medicine and Health, The University of Sydney, Sydney, New South Wales, Australia; 3 Facultad de Medicina, Universidad de Los Andes, Bogotá, Colombia; 4 Latin American Brain Health Institute (BrainLat), Universidad Adolfo Ibáñez, Santiago, Chile

**Keywords:** epidemiology, cohort studies, child health, birth weight, public health

## Abstract

**Background:**

It is hypothesised that lifelong physical activity behaviours are established in early life, however there is minimal, and contradictory, evidence examining prenatal and postnatal factors in relation to adulthood physical activity. We investigated associations between prospectively ascertained prenatal/postnatal factors and device-measured moderate-to-vigorous physical activity (MVPA) in midlife.

**Methods:**

Analyses included 5011 participants from the 1970 British Cohort Study, a birth cohort study of individuals born within the same week. At birth, the following factors were ascertained: socioeconomic position (SEP), maternal age, number of previous pregnancies, maternal smoking, maternal diabetes, gestational age, birth weight, breastfeeding status and infant health concerns. MVPA was captured at age 46 with a thigh-worn accelerometer device following a 24-hour protocol over 7 days.

**Results:**

In sex-adjusted models, lower SEP (−6.7 min/day (95% CI: −9.0 to –4.4) in those with a partly or unskilled paternal occupation), younger maternal age (0.4 min/day (0.2 to 0.5) per additional year of maternal age), maternal smoking during pregnancy (−2.5 min/day (−4.0 to –1.0)) and post-term gestational age (−7.4 min/day (−11.5 to –3.4); boys only) were associated with lower MVPA at age 46. In the mutually adjusted model, associations did not change but there was some evidence that birth weight may also be associated with MVPA levels.

**Conclusions:**

SEP, maternal age, maternal smoking, post-term birth in boys and birth weight were associated with MVPA in midlife, indicating that midlife physical activity behaviours may be partially established at birth. Early interventions in disadvantaged environments may have a positive impact on physical activity throughout the life course.

**WHAT IS ALREADY KNOWN ON THIS TOPIC**

- Current knowledge of the determinants of midlife physical activity has focused on factors in early and middle adulthood, with limited investigation of early life factors.
- Furthermore, the limited research in this area has often focused on retrospective recall of childhood factors and used self-reported measures of physical activity.

**WHAT THIS STUDY ADDS**

- This study identified several prenatal and postnatal factors, collected prospectively in 1970, that were associated with lower levels of device-measured moderate-to-vigorous physical activity: lower SEP, younger maternal age, smoking during pregnancy and post-term gestational age.

**HOW THIS STUDY MIGHT AFFECT RESEARCH, PRACTICE OR POLICY**

- Recognition that perinatal factors contribute to midlife activity has important implications for life-course approaches to policy and public health.
- Immediate and enduring physical behavioural activity interventions could target infants born into disadvantaged environments (eg, low SEP, younger mothers, unhealthy maternal behaviours and poorer health).

## Background

Physical inactivity is the fourth largest risk factor for mortality, responsible for an estimated 6%–8% of global deaths.^1^ There has been a substantial increase in physical activity research over the last half century,^2^ which have let to national activity guidelines^3 4^ and the implementation of WHO’s Global Action Plan for Physical Activity.^5^ Despite an increased focus in this area, available evidence suggests that worldwide trends in physical inactivity have not improved.^6^

Understanding early life determinants of physical (in)activity is an important first step in minimising the global inactivity pandemic. A series of umbrella systematic reviews have synthesised biological, psychological, behavioural, physical, sociocultural and socioeconomic determinants of physical activity across the life course.^7^ This evidence primarily assessed proximal factors from a single stage of life (eg, childhood factors related to childhood activity or adulthood factors related to adulthood activity). Physical activity patterns and behaviours in adulthood may be partially established in early life,^8^ potentially extending to intergenerational influences such as genetic heritability or parental health and behaviours.^9 10^ The idea that physical activity behaviours have developmental origins parallels the Developmental Origins of Health and Disease (DOHaD) hypothesis, originally proposed in the 1990s.^11^ DOHaD posits that exposure to environmental influences during critical developmental periods has a significant impact on adulthood chronic disease. Health behaviours, notably physical activity, may also originate in early life, providing one explanatory mechanism for persisting associations between early life factors and later life chronic disease.^12^

Studies investigating perinatal factors in relation to subsequent physical activity are sparse, with contradictory findings. For example, meta-analyses and multicohort analyses have reported positive,^13^ negative,^14 15^ U-shaped^16^ and null^17^ associations between birth weight and subsequent physical activity levels. The contradictory nature of this evidence extends to other factors such as socioeconomic position (SEP)^14 18 19^ and gestational age.^14 15 20^ While differences may partially be explained by country-level socioeconomic factors, the existing evidence has many limitations. Studies have focused nearly entirely on birth weight or SEP,^13 16–20^ with little consideration of other perinatal aspects related to infant health or maternal characteristics. Early life factors are often ascertained by retrospective recall,^13 17–19^ while device-measured physical activity ascertainment is uncommon. This is concerning as accuracy of physical activity recall differs by SEP^21^; therefore, the use of self-reported data may confound this association. Finally, most studies have considered physical activity in childhood, adolescence or early adulthood, with—to our knowledge—no investigation of how such associations may extend to midlife and later life.

Early identification of physical inactivity risk factors is crucial to facilitate interventions that can minimise the sequelae of age-related health outcomes associated with physical inactivity. The 1970 British Cohort Study (BCS70), a population-representative birth cohort, is uniquely positioned to examine this question due to the prospective ascertainment of perinatal factors and the collection of device-measured moderate-to-vigorous physical activity (MVPA) data at age 46. The aim of this study was to investigate associations between prospectively ascertained prenatal and postnatal factors and device-measured MVPA in midlife.

## Methods

### Study sample

**Figure 1:**
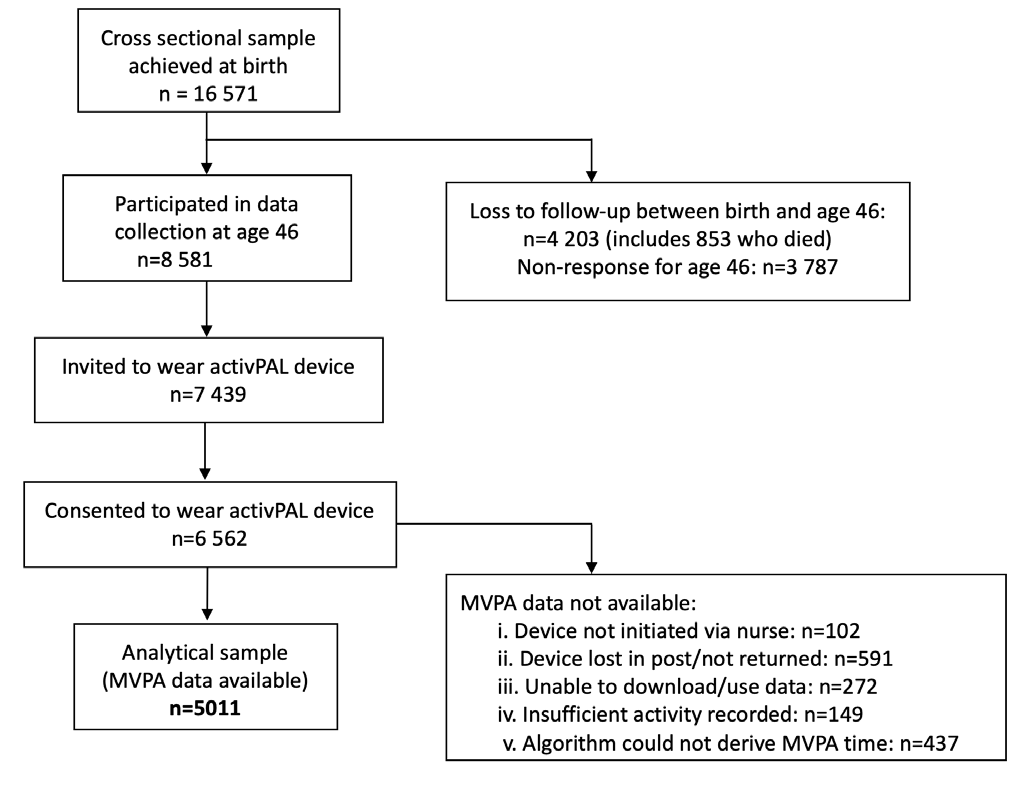
Flow chart indicating derivation of analytical sample (n=5011). MVPA, moderate-to-vigorous physical activity.

The BCS70 is a study of 16 571 individuals born in England, Scotland and Wales within 1 week of another in April 1970. Participation rates and characteristics of those lost to follow-up have been detailed elsewhere.^22^ Initial data collection occurred within the first week of birth; midwives completed a confidential survey by interviewing the mother and doctor and reviewing clinical records. The most recent data collection occurred at age 46 and included an interview, questionnaire, clinical assessments and a 24-hour, 7-day accelerometer wear protocol. Individuals were included if they had information on one or more factors collected at birth and on MVPA levels at age 46. See Figure (’Figure’ and ‘Table’ capitalisation is inconsistent) figure 1 for derivation of the study sample (n=5011). Study data are available from UK Data Service.^23 24^

### Early life exposures

Prenatal and postnatal factors were chosen a priori based on previous evidence and hypotheses of associations with physical activity, health or health behaviours.^13 16–20^ Table 1 outlines all nine factors including measurement and analytical coding details. This includes parental occupational class, prenatal maternal factors (age at birth, previous pregnancies, maternal smoking during pregnancy and clinical diabetes) and postnatal infant factors (gestational age, birth weight, breast feeding and infant health concerns). Note that correlations between factors were all <0.15, with the exception of correlations between maternal age and previous pregnancies (0.51) and birth weight and gestational age (0.32).

**Table 1:** Descriptive characteristics of individuals included in analyses (n=5011)*

Prefactor or postfactor	Details of measurement in 1970	Coding framework for analysis
1. Parental occupational class	Father’s occupation at birth using the Registrar General’s 1966 Classification of Occupation; if missing, maternal occupational class was used.	Categorical (I—professional or II—managerial/technical; III—manual or non-manual; IV—partly skilled or V-unskilled)
**Prenatal maternal factors**		
2. Maternal age at birth	Calculated using the mother and child’s dates of birth.	Continuous (years)
3. Previous pregnancies	Calculated from detailed reporting of all previous pregnancies at the time of the cohort members’ birth, including miscarriages and ectopic pregnancies.	Categorical (0, 1–2, 3+)
4. Smoking during pregnancy	Reported yes or no to midwife interview.	Categorical (yes/no)
5. Clinical diabetes	Reported by midwives at birth.* Due to potential under-reporting of diabetes at birth, this factor was supplemented with additional data collected at age 5, where a health visitor examined all home visit records, consultation record cards, family records, child health clinics and child welfare clinic records and reported if diabetes was noted as a risk factor during the perinatal period (pregnancy, labour or postnatal in first week). Note that details (eg, type 1, type 2, gestational, etc) were not collected.	Categorical (yes/no)
**Postnatal infant factors**		
6. Gestational age	Calculated from the reported first day of the last menstrual period to birth. The data processing team in 1970 coded ‘implausible’ values (after consideration of date of last delivery, duration of gestation, birth weight) as missing.	Categorical (preterm <37 weeks; term 37–41 weeks; post-term >41 weeks)
7. Birth weight	Recorded in pounds or grams.	Continuous (kg)
8. Breast feeding	At the age 5 home visit, mothers were asked if, and for how long, the study member was partly or wholly breast fed.	Categorical (0–3 months; 3+ months)
9. Infant health concerns	At birth, nurses recorded if the baby had any of the following binary health indicators or interventions: jaundice, breathing difficulties including respiratory distress syndrome, cyanotic attacks, fits or convulsions, cerebral signs, fractures, cephalhaematoma, sticky eyes, discharge form umbilicus, received exchange transfusions, underwent an operation or had any other illness/condition.	Categorical (0, 1, 2+)

*Question: ‘Has the mother clinical diabetes?’

### Moderate-to-vigorous physical activity

MVPA was measured using the activPAL3 micro (PAL Technologies, Glasgow, UK), a thigh-worn accelerometer device. The wear protocol is described in detail elsewhere.^25^ Briefly, research nurses fitted the device on the midline of participant’s upper thigh. Participants were instructed to wear it continuously for seven full days including during sleeping, bathing and swimming activities. Default settings were used for initialisation of devices and data were processed at 1 s intervals using previously validated open source software that isolates valid awake wear time from sleep and prolonged non-wear periods.^26^ Participants were included in analyses if they had 1+ valid day of wear time (10+ hours of waking wear time over each midnight-to-midnight period). Although 62% of all wearers had a full 7 days of wear time, all models were adjusted for wear time. A step cadence threshold of ≥100 steps/min was used to estimate time spent in MVPA,^27^ which was expressed as the average minutes per day. Figure 1 outlines accelerometer participation rates.

### Statistical analyses

Differences in sample characteristics between males and females were compared using chi square tests and one-way analysis of variances (ANOVAs). Linear regressions examined associations between prenatal and postnatal factors with MVPA in three stages. First, individual models considered the association between each factor and MVPA, adjusting for sex and wear time. Interactions between sex and each factor were assessed and deviation from linearity was also tested for the two continuous variables (ie, maternal age, birth weight). Next, each model was additionally adjusted for SEP at birth to assess if prenatal and postnatal factors were independently associated with MVPA or explained by SEP. Finally, all factors were included in a single mutually adjusted model.

Missing data ranged from 7.3% (previous pregnancies) to 23.6% (gestational age). Therefore, following a missing at random assumption, covariate data were imputed using multiple imputation chained equations in Stata V.17. All prenatal and postnatal factors, sex, MVPA and wear time were included in the imputation model. Based on the fraction of missing information, estimates from 25 imputed datasets were combined using Rubin’s rules. Sensitivity analyses replicated the models in a subsample of those with complete data on all factors (n=3209). To assess the impact of gestational age on birth weight, we repeated the sex-adjusted and wear time-adjusted and the sex, wear time and SEP models while including both birth weight and gestational age in each interim model. To further investigate the impact of missing data, differences in early life factors were assessed among those included in the analytical sample (n=5011), those who participated at age 46 but did not have MVPA data (n=3570) and those who were lost to follow-up before age 46 (n=9425) using χ^2^ test, one-way ANOVA and post hoc test.

## Results

There were minimal differences between males and females across most early life factors (see table 2), although males weighed more (3382 g±534 g vs 3255 g±498 g) and were more likely to have 1+ infant health concern (27.1% vs 21.8%). At age 46, males had an average MVPA time of 50.4±26.6 min/day, which was similar to females (51.6±24.7 min/day). However, males had slightly higher wear time (16.0±1.3 hours/day vs 15.7±1.3 hours/day). As there was no evidence of an interaction between sex and eight of nine early life factors, males and females were included in the same model. There was an interaction term between sex and post-term gestational age, which was included in any model containing gestational age and estimates for males and females are provided separately.

**Table 2:** Descriptive characteristics of individuals included in analyses (n=5011)*

	Males (n=2396, 47.8%)	Females (n=2615, 52.2%)	Missing (n, %)
Parental occupational class, n (%)			
I—Professional, II—managerial/technical	479 (21.8)	520 (21.4)	391 (7.8)
III—Manual or non-manual	1311 (59.8)	1419 (58.5)	
IV—Partly skilled, V—unskilled	404 (18.4)	487 (20.1)	
Prenatal maternal factors			
Age at birth, mean (SD)	26.0 (5.3)	26.0 (5.3)	372 (7.4)
Previous pregnancies, n (%)			366 (7.3)
0	806 (36.6)	901 (36.9)	
1–2	1070 (48.6)	1194 (48.9)	
3+	325 (14.8)	349 (14.3)	
Smoking during pregnancy, n (%)	917 (42.0)	1069 (44.0)	395 (7.9)
Clinical diabetes, n (%)	19 (0.9)	20 (0.8)	401 (8.0)
Postnatal infant factors			
Gestational age, n (%)			1182 (23.6)
Preterm (<37 weeks)	73 (4.0)	79 (3.9)	
Term (37–41 weeks)	1586 (87.5)	1736 (86.1)	
Post-term (>41 weeks)	154 (8.5)	201 (10.0)	
Birth weight (g), mean (SD)	3382 (534)	3255 (498)	368 (7.3)
Breast feeding >3 months, n (%)	262 (13.5)	291 (13.5)	907 (8.6)
Infant health concerns, n (%)			431
0	1578 (72.9)	1890 (78.2)	
1	474 (21.9)	445 (18.4)	
2+	112 (5.2)	81 (3.4)	
Accelerometry data			
MVPA minutes, mean (SD)	50.4 (26.6)	51.6 (24.7)	0 (0)
Wear time hours, mean (SD)	16.0 (1.3)	15.7 (1.3)	0 (0)

*Sample size for each prenatal/postatal factor varies due to missing data.

MVPA, moderate-to-vigorous physical activity.

**Figure 2:**
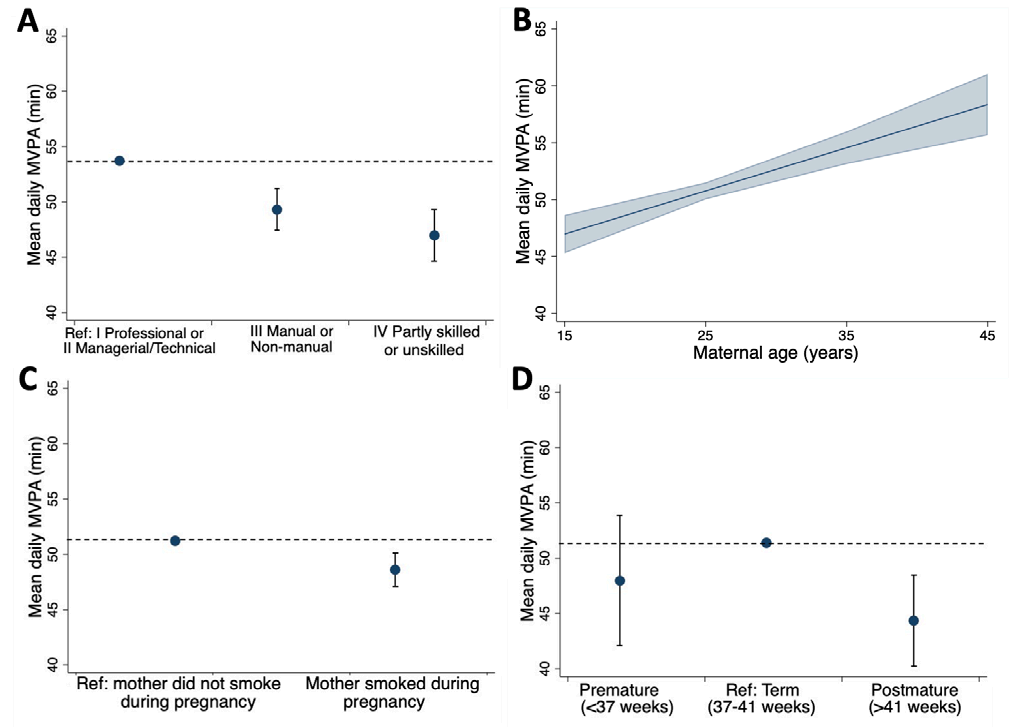
Associations between (A) socioeconomic position at birth, (B) maternal age, (C) maternal smoking during pregnancy, (D) gestational age (boys only) and moderate-to-vigorous physical activity (MVPA) at age 46 in sex-adjusted and wear time-adjusted model (CIs indicated with error bars or shading)

There was no evidence of a non-linear association between either maternal age or BMI with MVPA. In sex and wear time-adjusted models, lower SEP, younger maternal age, maternal smoking during pregnancy and post-term gestational age were associated with lower MVPA levels at age 46 (table 3). Individuals whose parental occupational class was categorised as ‘IV—partly skilled/V—unskilled’ or as ‘III—manual/non-manual’ had 6.67 (95% CI: −8.95 to –4.38) and 4.33 (−6.16 to –2.50) fewer daily MVPA minutes than those with parents with ‘I—professional’ or ‘II—managerial/technical’ occupations (figure 2A). Each additional year of maternal age at birth was associated with 0.38 (95% CI: 0.24 to 0.52) higher MVPA minutes per day (figure 2B). Those whose mothers smoked during pregnancy partook in 2.52 (95% CI: −4.02 to –1.02) fewer MVPA minutes per day (figure 2C). Compared with males born at term, males born post-term (>41 weeks) averaged 7.43 (95% CI: −11.45 to –3.40) fewer daily MVPA minutes, although there was no association in females (figure 2D). Estimates for each of these factors remained after adjustment for SEP. In a mutually adjusted model of all factors, associations of SEP, maternal age, maternal smoking and post-term males with MVPA remained, with minimal attenuation of effect sizes (table 3). Additionally, there was some evidence to indicate that higher birth weight may be associated with lower MVPA in the adjusted model (β=−1.51 (95% CI: −3.07 to 0.05) min/day per 1 SD).

**Table 3:** Association between early life risk factors and time spent in MVPA at age 46 (β (95% CI); change in mean daily MVPA per 1 unit/category change in risk factor, n=5011)

	Adjusted for sex and wear time*		Adjusted for sex, wear time, SEP†		Mutually adjusted model‡	
Parental occupational class						
I—Professional, II—managerial/technical	Ref	<0.001	--		Ref	<0.001
III—Manual or non-manual	–4.33 (–6.16 to –2.50)				–3.56 (–5.47 to –1.65)	
IV—Partly skilled, V—unskilled	–6.67 (–8.95 to –4.38)				–5.43 (–7.84 to –3.03)	
Prenatal maternal risk factors						
Maternal age at birth, per 1 year	0.38 (0.24 to 0.52)	<0.001	0.33 (0.19 to 0.47)	<0.001	0.30 (0.14 to 0.46)	<0.001
Previous pregnancies						
0	Ref	0.15	Ref	0.10	Ref	0.95
1–2	1.18 (–0.41 to 2.77)		1.10 (–0.48 to 2.68)		0.26 (–1.40 to 1.92)	
3+	2.05 (–0.24 to 4.34)		2.40 (0.13 to 4.69)		0.30 (–2.30 to 2.89)	
Smoking during pregnancy	−2.52 (–4.02 to –1.02)	<0.001	−1.98 (–3.50 to –0.46)	<0.05	−1.93 (–3.46 to –0.39)	0.01
Clinical diabetes	2.84 (–5.24 to 10.92)	0.49	2.73 (–5.32 to 10.77)	0.51	1.59 (–6.49 to 9.66)	0.70
Postnatal infant risk factors						
Gestational age						
Males						
Preterm (<37 weeks)	−3.69 (–9.69 to 2.31)	<0.005	−3.56 (–9.54 to 2.43)	<0.005	−4.01 (–10.36 to 2.34)	<0.001
Term (37–41 weeks)	Ref		Ref		Ref	
Post-term (>41 weeks)	−7.43 (–11.45 to –3.40)		−7.05 (–11.10 to –3.01)		−6.28 (–10.48 to –2.08)	
Females						
Preterm (<37 weeks)	−2.75 (–8.52 to 3.01)	0.47	−2.75 (–8.50 to 3.01)	0.51	−3.60 (–9.09 to 1.88)	0.37
Term (37–41 weeks)	Ref		Ref		Ref	
Post-term (>41)	−1.57 (–5.22 to 2.07)		−1.39 (–5.02 to 2.23)		−0.85 (–4.67 to 2.38)	
Birth weight, per 1 kg	−0.36 (–1.81 to 1.08)§	0.62	−0.61 (–2.05 to 0.83)¶	0.41	−1.51 (–3.07 to 0.05)	0.06
Breast feeding >3 months, n (%)	2.37 (0.02 to 4.73)	0.05	1.46 (–0.90 to 3.82)	0.22	0.84 (–1.58 to 3.26)	0.50
Infant health concerns, n (%)						
0	Ref	0.42	Ref	0.47SS	Ref	0.47
1	–0.37 (–2.23 to 1.48)		–0.34 (–2.19 to 1.52)		–0.39 (–2.31 to 1.52)	
2+	–2.45 (–6.15 to 1.25)		–2.26 (–5.94 to 1.42)		–2.34 (–6.12 to 1.43)	

*Nine individual models, each adjusted for sex and wear time.

†Eight individual models, each adjusted for sex, wear time and paternal occupational class.

‡A single combined model including sex, wear time, paternal occupational class, maternal age at birth, previous pregnancies, maternal smoking during pregnancy, maternal clinical diabetes, gestational age, standardised birth weight, breast feeding and infant health concern.

§Estimate for birth weight in model adjusted for sex and gestational age: −0.63 (95% CI: −2.21 to 0.94); p=0.43.

¶Estimate for birth weight in model adjusted for sex, wear time, SEP and gestational age: −0.91 (95% CI: −2.48 to 0.66); p=0.26.

MVPA, moderate-to-vigorous physical activity; Ref, reference; SEP, socioeconomic position.

### Additional analyses

Associations remained largely the same when analyses were restricted to individuals with complete data (n=3209; online supplemental eTable 1). There were some minor differences in the mutually adjusted model; specifically, the relationship between SEP and MVPA weakened, with larger associations for smoking and birth weight. For example, a 1 kg increase in birth weight was associated with 2.61 (95% CI: −4.47 to –0.74) fewer daily MVPA minutes. Initial associations between birth weight and MVPA did not change when gestational age was included in the interim model (table 3, online supplemental eTable 1).

10.1136/jech-2022-219213.supp1 Supplementary data

There were differences between those included in the analytical sample (n=5011) and both those who were lost to follow-up before age 46 (n=9425) and those who participated in the age 46 collection but did not have MVPA data (n=3570). The analytical sample were more likely to be female and advantageous prenatal and postnatal factors including: higher SEP, gestational age between 37 and 41 weeks, a mother who did not smoke during pregnancy, were breast fed for 3+ months, higher birth weight and fewer infant risk factors (online supplemental eTable 2). Post hoc tests revealed that differences were largest between the analytical sample and those lost to follow-up before age 46.

## Discussion

This study identified consistent associations between several prenatal and postnatal factors and midlife MVPA. There was a positive association of higher parental SEP and older maternal age at birth with MVPA at age 46. Additionally, those whose mothers smoked during pregnancy and post-term boys (ie, gestational age >41 weeks) were more likely to partake in lower levels of MVPA. Associations were not explained by adjustment for other early life factors. These novel findings build on existing evidence demonstrating that early life factors influence physical activity behaviours in childhood, adolescence and early adulthood,^13–16^ by showing associations remain in midlife.

### Comparison with other studies

Studies have reported contradictory associations of early life SEP, birth weight and gestational age with physical activity in early adulthood. Most systematic reviews have reported that lower childhood SEP is associated with lower physical activity in adulthood, with the strongest evidence from high-income countries and for leisure time-specific physical activity.^18 19^ The evidence presented here supports this association. However, some studies have reported an opposite pattern,^18 19^ and recent evidence from three Brazilian birth cohorts suggests higher SEP at birth is associated with lower device-measured MVPA at ages 6, 18 and 30.^14^ With heterogeneity in physical activity assessment method (eg, self-reported, device-measured), activity domain (eg, leisure time, occupation, transport), country and age, it is not possible to identify a single explanation for these divergent findings.^21 28^

There is also inconsistent evidence pertaining to birth weight and gestational age. We identified a weak linear association between high birth weight and low MVPA, which has been previously reported in meta-analyses and cross-cohort studies^14 15^ alongside inverse U-shaped^13 16^ and null associations.^17^ Ding *et al* 14 reported no association between gestational age and MVPA at ages 6, 18 or 30, whereas Tikanmäki *et al* 15 reported that those born preterm or post-term had lower levels of leisure-time physical activity at age 16 than regular term births. Here, we present the novel finding that post-term boys have lower levels of physical activity in midlife, with no association in girls. Finally, there is limited evidence examining maternal factors related to offspring physical activity behaviours, and thus comparison with other studies is limited. To our knowledge, studies have not previously investigated maternal age or diabetes. However, there is some evidence, including in BCS70, that maternal health may contribute to physical activity as greater maternal BMI has been linked to lower levels of physical activity and cardiorespiratory fitness in offspring.^15 29^

### Mechanisms of associations

Associations may act via multiple pathways; an environmental-social pathway and a biological pathway are described below. Despite independent associations of parental SEP, maternal age and maternal smoking with MVPA, these early life factors may share a common environmental-social pathway. Higher SEP and older maternal age may positively contribute to a healthier early life environment for offspring. Specifically, older maternal age is a major determinant of greater health literacy, which can directly shape health behaviours in children,^30^ while higher SEP at birth is linked to better nutrition and higher physical activity in childhood, adolescence and adulthood.^31^ Considered alongside previous evidence reporting associations between early life factors and physical activity levels in childhood and early adulthood,^13–16^ it is likely that early life factors contribute to activity habits at earlier ages which are then sustained into midlife and later life. Further consideration of how associations change across time is needed. Maternal smoking may also be an indicator of parental health behaviours during childrearing, impacting the child’s own health behaviours and decision making.^32^ For example, higher maternal BMI (measured at age 10) in BCS70 is associated with more sedentary and less MVPA time at age 46, although it is noteworthy that there were no associations with paternal BMI.^29^ Finally, SEP tracks over the life course, such that children with higher early life SEP are more likely to have higher SEP in adulthood.^12^ Greater adulthood SEP in adulthood is linked to higher non-occupational and leisure-time physical activity participation.^33^

The finding that post-term boys have lower MVPA levels in midlife indicates a sex-specific biological pathway. Post-term births increase risk of poor childhood health (eg, respiratory distress, fetal macrosomia, dysmaturity syndrome, poorer physical function^34^), which can directly contribute to lower engagement in higher intensity activities in both school and extracurricular settings.^35^ Additionally, oxygen deficiency and abnormal neuroendocrine environment in post-term pregnancies may contribute to increased risk of behavioural and emotional problems,^36^ which are more common in boys and linked to physical inactivity.^37^ Males born post-term also have higher risk of obesity in adolescence, which is both affected by and subsequently to low levels of physical activity throughout the life course.^38 39^ It is difficult to disentangle whether the risk associated with longer gestational age is due to longer exposure to the prenatal environment or if the extended gestational period is a response to ill health of the infant or mother during the normal gestational period. With the modern obstetric practice of inducing mothers to reduce late-term delivery risks, this is a challenging, yet worthwhile, mechanism to disentangle. Finally, maternal factors including high BMI, hormone deficiency and genetic factors can directly contribute to increased risk of post-term births.^34^ Therefore, there may be a cumulative effect of both the biological and environmental-social pathway on physical activity levels. Further research should investigate mediating pathways using data from childhood through to early adulthood to test the environmental-social biological pathways, hypothesised above.

### Strengths and limitations

The main strengths of this study relate to the ascertainment of exposures and the MVPA outcome. Early life factors were collected immediately after birth using clinical records and interviews with the mother and doctor, minimising recall bias or inaccuracies in subjective reporting. MVPA was ascertained using a 24-hour accelerometer protocol to accurately capture all activity across the wear period.^25^ The large age-homogenous and population-representative sample is an important strength of the study as it eliminates age-related confounding, which can be extensive when considering how physical activity behaviours change with age.

There are some limitations. Those lost to follow-up between birth and age 46 were more likely to have poorer health at birth and in midlife,^25^ however we hypothesise effect sizes may be underestimated by this loss to follow-up bias as those with disadvantaged circumstances at birth were less likely to have MVPA data at age 46. The quality of risk factor data is influenced by historical limitations relevant to the 1970 ascertainment. For example, there was limited data on infant health, thus an aggregate sum of any abnormal conditions was used as a proxy for overall health in first week of life. Additionally, binary ascertainment of some self-reported variables was necessary due to data collection methods (eg, breast feeding, smoking during pregnancy), and thus dose-response associations could not be estimated. There were high levels of missing data among some risk factors; this was driven primarily by gestational age. Ascertainment of gestational age relied on recall of last menstrual cycle and, therefore, is less accurate than ultrasounds used today. It is likely that maternal diabetes was under-reported, due to a historical unawareness on screening and diagnosis of gestational diabetes, which may have resulted in insufficient power to identify an association. The BCS70 birth sweep focused on the infant and mother, with little consideration of paternal factors beyond occupational class; strong overlap of maternal and paternal age, smoking habits and previous children is expected and therefore interpretation of maternal risk factors must proceed with caution.^40^ Finally, MVPA was assessed at a single age. Although there is inconsistent tracking of physical activity behaviours across the life course, future research should consider how these patterns extend to other ages including adolescent, early and late adulthood. Additional research must now consider specific mediation pathways, such as body composition, lifelong SEP and occupational activity, to identify meaningful opportunities for interventions.

## Conclusions

This study adds to the evidence base of early programming of physical activity by demonstrating that associations between prenatal and postnatal factors and physical activity extend beyond childhood, adolescence and early adulthood and are maintained into midlife.

Consistent with the DOHaD paradigm,^11^ our findings suggest that physical activity behaviours may be partially determined by factors emerging as early as birth. This complements a life course approach to chronic disease in adulthood by demonstrating a plausible behavioural pathway through which some of these associations may occur. The effect sizes presented here (eg, as much as 7+ min fewer daily MVPA minutes in post-term boys compared with normal term) are not trivial. Evidence suggests that 10 additional minutes of daily MVPA is associated with an 8%–11% reduction in cardiovascular disease risk^41 42^ and a 6%–38% reduction in mortality risk,^43 44^ while current physical activity recommendations advocate for 10–30 min of daily MVPA.^3 4^ Therefore, immediate public health implications must consider how infants born into disadvantaged environments—including low SEP, young mothers, unhealthy maternal behaviours (eg, smoking) and poorer health (eg, higher birth weight, post-term births)—can be identified for earlier interventions targeting physical activity health behaviours.
