# Supplementary material for: Prenatal and postnatal correlates of moderate-to-vigorous physical activity in midlife: evidence from the 1970 British Cohort Study
Source: J Epidemiol Community Health. 2022 Sep 9;76(11):949–55. doi: 10.1136/jech-2022-219213 (PMC9554029; doi:10.1136/jech-2022-219213)
Supplement: Supplementary data [file jech-2022-219213supp001.pdf]

eTable 1. Linear regression detailing change in MVPA in restricted complete cases sample (n=3 209)

	**Adjusted for sex and wear-time [a]**		**Adjusted for sex, wear-time, SEP [b]**		**Mutually-adjusted model [c]**	
**PARENTAL OCCUPATIONAL CLASS**						
I Professional, II Managerial/Technical	Ref		--		Ref	
III Manual or Non-manual	-3.17 (-5.34, -1.00)	<0.005			-2.13 (-4.34, 0.07)	0.10
IV Partly skilled, V unskilled	-4.36 (-7.19, -1.53)				-2.86 (-5.78, 0.05)	
**PRE-NATAL MATERNAL RISK FACTORS**						
Maternal age at birth, per 1 year	0.34 (0.17, 0.51)	<0.001	0.31 (0.14, 0.48)	<0.001	0.28 (0.08, 0.48)	<0.01
Previous pregnancies						
0	Ref		Ref		Ref	
1-2	1.14 (-0.77, 3.05)	0.32	1.14 (-0.77, 3.04)	0.25	0.51 (-1.48, 2.51)	0.25
3+	1.88 (-0.86, 4.63)		2.17 (-0.57, 4.91)		0.55 (-2.59, 3.69)	
Smoking during pregnancy	-3.15 (-4.92, -1.37)	<0.001	-2.84 (-463, -1.05)	<0.005	-2.92 (-4.76, -1.09)	<0.005
Clinical diabetes	-5.35 (-15.5, 4.83)	0.30	-5.32 (-15.49, 4.84)	0.31	-5.71 (-15.84, 4.42)	0.27
**POST-NATAL INFANT RISK FACTORS**						
Gestational age						
**BOYS**						
Pre-term (<37 weeks)	-2.23 (-8.73, 4.26)		-2.04 (-8.53, 4.45)		-3.49 (-10.27, 3.29)	
Term (37-41 weeks)	Ref	<0.005	Ref	<0.005	Ref	<0.005
Post-term (>41 weeks)	-8.30 (-12.83, -3.77)		-8.00 (-12.53, -3.47)		-7.28 (-11.81, -2.76)	
**GIRLS**						
Pre-term (<37 weeks)	-3.81 (-10.30, 2.67)		-2.91 (-16.15, 10.33)		-4.82 (-11.53, 1.88)	
Term (37-41 weeks)	Ref	0.35	Ref	0.42	Ref	0.65
Post-term (>41 weeks)	-1.71 (-5.59, 2.17)		-1.59 (-11.40, 8.21)		-1.00 (-4.88, 2.88)	
Birth weight, per 1kg	-1.25 (-2.96, 0.46)[d]	0.15	-1.38 (-3.09, 0.33)[e]	0.11	-2.61 (-4.47, -0.74)	<0.01
Breastfeeding >3months, n(%)	2.91 (0.39, 5.43)	0.02	2.36 (-0.19, 4.90)	0.07	1.62 (-0.93, 4.18)	0.21
Infant health concerns, n(%)						
0	Ref		Ref		Ref	
1	0.08 (-2.12, 2.27)	0.61	0.13 (-2.07, 2.32)	0.67	-0.27 (-2.49, 1.95)	0.57
2+	-2.27 (-6.83, 2.29)		-2.03 (-6.59, 2.52)		-2.53 (-7.23, 2.17)	

[a] 9 individual models, each adjusted for sex and wear time
[b] 8 individual models, each adjusted for sex, wear time and paternal occupational class
[c] A single combined model including sex, wear time, paternal occupational class, maternal age at birth, previous pregnancies, maternal smoking during pregnancy, maternal clinical diabetes, gestational age, standardised birth weight, breastfeeding and infant heath concerns
[d] Estimate for birth weight in model adjusted for sex and gestational age: -1.57 (-3.39, 0.24); p=0.09
[e] Estimate for birth weight in model adjusted for sex, wear time, SEP and gestational age: -1.69 (-3.50, 0.12); p=0.07

eTable 2. Comparison of pre- and post-natal factors between the analytical sample (n=5 011), those who participated in age 46 data collection but did not have MVPA data (n=3 570) and those who were lost to follow-up before age 46 (n=9 425)

	**Analytical sample** (n=5 011)	**Participated in age 46 data collection but no MVPA data** (n=3 570)	**Lost to follow-up before age 46** (n=9 425)
**SEX, n(%)**			
Male	2396 (47.8)	1762 (49.4)	5087 (55.1)
Female	2615 (52.2)	1808 (50.6)	4147 (44.9)
**PARENTAL OCCUPATIONAL CLASS, n(%)**			
I Professional, II Managerial/Technical	999 (21.6)	683 (20.7)	1428 (15.6)
III Manual or Non-manual	2730 (59.1)	1966 (59.7)	5245 (57.3)
IV Partly skilled, V unskilled	891 (19.3)	645 (19.6)	2474 (27.1)
**PRE-NATAL MATERNAL RISK FACTORS**			
Age at birth, mean ±SD	26.0 ±5.3	25.9 ±5.4	26.0 ±5.7
Previous pregnancies, n(%)			
0	1707 (36.8)	1138 (34.4)	2975 (32.2)
1-2	2264 (48.7)	1609 (48.7)	4283 (46.3)
3+	674 (14.5)	559 (16.9)	1987 (21.5)
Smoking during pregnancies, n(%)	1986 (43.0)	1443 (43.9)	4470 (48.6)
Clinical diabetes, n (%)	39 (0.9)	26 (0.8)	65 (0.7)
**POST-NATAL INFANT RISK FACTORS**			
Gestational age: males, n(%)			
Pre-term (<37 weeks)	73 (4.0)	70 (5.2)	315 (8.1)
Term (37-41 weeks)	1586 (87.5)	1138 (84.7)	3184 (81.8)
Post-term (>41 weeks)	154 (8.5)	135 (10.1)	393 (10.1)
Gestational age: females, n(%)			
Pre-term (<37 weeks)	79 (3.9)	51 (3.7)	225 (7.1)
Term (37-41 weeks)	1736 (86.1)	1195 (85.6)	2628 (83.1)
Post-term (>41 weeks)	201 (10.0)	146 (10.5)	309 (9.8)
Birth weight (g), mean ±SD			
Males	3382 ± 534	3361 ±522	3282 ±0.65
Females	3255 ± 498	3257 ±514	3168 ±598
Breastfeeding >3months, n(%)	553 (13.5)	349 (12.2)	510 (8.5)
Infant health concerns, n(%)			
0	3468 (75.7)	2432 (74.6)	6555 (74.1)
1	919 (20.1)	687 (21.1)	1803 (20.4)
2+	193 (4.2)	143 (4.4)	487 (5.51)

[a] N varies due to missing data in early life factors
